# Rheology of Biopolymer Solutions and Gels

**DOI:** 10.1100/tsw.2003.15

**Published:** 2003-03-24

**Authors:** David R. Picout, Simon B. Ross-Murphy

**Affiliations:** Biopolymers Group, Division of Life Sciences, King's College London,London, UK

**Keywords:** rheology, stress and strain, biopolymers, green polymers, polysaccharides, proteins, solutions and gels, oscillatory measurements, viscoelastic behaviour, entanglement networks, small and large deformations, critical gels, weak gels

## Abstract

Rheological techniques and methods have been employed for many decades in the characterization of polymers. Originally developed and used on synthetic polymers, rheology has then found much interest in the field of natural (bio) polymers. This review concentrates on introducing the fundamentals of rheology and on discussing the rheological aspects and properties of the two major classes of biopolymers: polysaccharides and proteins. An overview of both their solution properties (dilute to semi-dilute) and gel properties is described.

## FUNDAMENTALS OF RHEOLOGY

The term 'rheology' is derived from the Greek word *rheos* meaning to stream or flow. Rheology is the study of the flow and deformation of materials, and describes the response of different materials. For example, ideal solids deform elastically (Hookean response), whereas liquids flow. As we see below most solid materials will behave as liquids at very long times and most liquids will respond like solids at short times. They cover the gamut of “viscoelastic materials”. As far as biopolymers are concerned, they occur usually as liquids in solution, although some can be coaxed to form soft solids (gels). This article describes the rheological techniques for characterizing biopolymer solutions and gels.

## BIOPOLYMER SYSTEMS

The major classes of biopolymer are proteins, nucleic acids, and polysaccharides. In the present article we will not discuss nucleic acids, because the amounts usually available are not large, and rheology as a technique tends to be rather sample intensive (typically ~20 mg or more is needed for a single experiment). Consequently we will mainly discuss proteins and polysaccharides. Again because many are globular and, as we shall see later, the rheological properties (say viscosity) of a polymer in solution depend upon the “swept out” molecular volume, protein solutions are studied relatively little. However, both cold- and heat-set protein gels can be prepared and we discuss this later. Finally, polysaccharides have been widely studied; they are usually of high molecular weight (say $>\sim 1 \times 10^6$) and semi-flexible, so they occupy a large volume in solution. This, in turn, means that most contribute very significantly to solution viscosity, and some of these are gel formers.

The other factor is that they are available in very large quantities compared to most proteins, and three of them (cellulose, starch, and chitin) are amongst the commonest materials in the biosphere.

### Solid Materials

The stress on a material is defined as the force F acting per initial unit area $A_0$. This tends to produce a deformation or strain defined as the ratio of the change in dimension relative to the original dimensions. For a tensile deformation the strain is dl/l, where l is the original dimension (length) and dl the change produced by application of the stress. For shear stress the tangent of the angle α gives the shear strain, which is also dl/l. For small strains tan α ≈ α. the Young's or tensile modulus is then given by:

$$E = \varepsilon / \alpha = (F / A_0)/(dl / l) \quad (1)$$

(Since $A_0$ will tend to change when the sample is deformed, the true stress is sometimes defined as F/A, the area at the given strain), and correspondingly the shear modulus may be written as follows:

$$G = \tau / \gamma = (F / A_0)/\tan\alpha \qquad \text{if } \tan\alpha \cong \alpha \quad \therefore G \cong F/(A_0\alpha) \quad (2)$$

Strain is dimensionless, and the SI units of modulus are $Nm^{-2}$ or Pa. (The CGS unit of modulus is dynes $cm^{-2}$ [to convert from dynes $cm^{-2}$ to Pascals divided by ten]). In practice, a 'geometry' such as the parallel plate or cone/plate is used (Fig. 1). Here we have implied that the stress is applied to the sample and the strain is measured as a change in dimensions or a displacement. It is often as useful to apply a known deformation and measure the force. Since stress and strain are closely related, e.g., by Eq. 2, it is only important to know which is the dependent and which is the independent "variable".

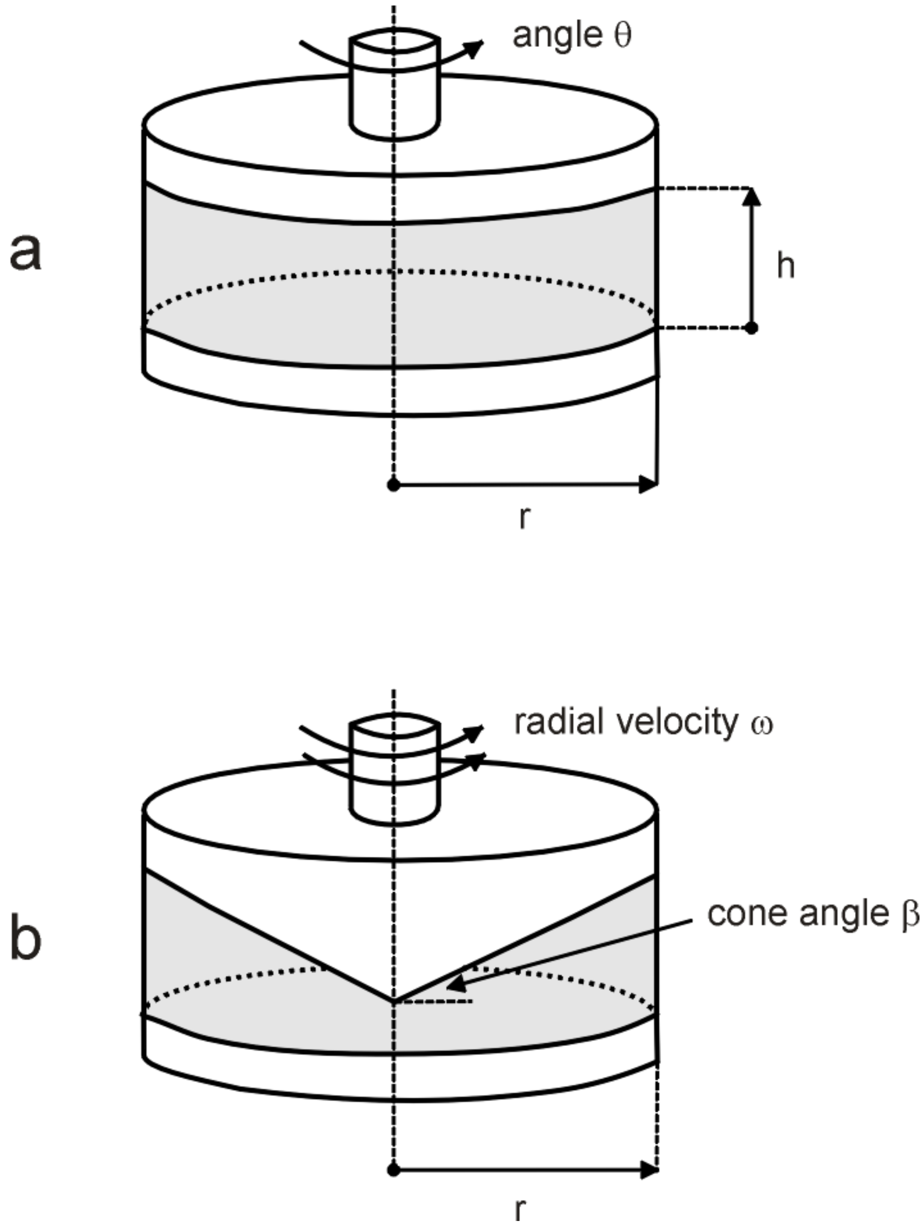

**FIGURE 1.** Parallel plate and cone/plate geometries.

## Fluid Materials

The definitions above are correct for an ideal elastic (Hookean) solid for all stresses, strains, and times. For fluids, the stress depends upon the rate of change of strain with time rather than the amount of deformation. The shear viscosity is defined by:

$$\eta = \tau / (d\gamma / dt) = \tau / \dot{\gamma} \qquad (3)$$

Where $\dot{\gamma}$ is the shear strain rate (or shear rate). The corresponding SI unit of viscosity is the Pa s; the viscosity of water at 20°C is almost exactly $10^{-3}$ Pa s. The shear rate in the cone/plate is constant across the gap and is given by $\dot{\gamma} = \Omega/\beta$, where $\Omega$ is the radial velocity of rotation (radian $s^{-1}$) and the cone angle $\beta$ is measured in radians. Corresponding to the shear viscosity the extensional (elongational) viscosity can also be defined by $\overline{\eta}\ (= \sigma / \dot{\varepsilon})$.

The above definitions are rigorous but they have only a limited applicability. This is because the ratio of stress to strain for solids and liquids is independent of strain and constant (independent of strain rate) only as $\gamma\,(\dot{\gamma}) \to 0$. In general there are many forms of stress-strain curves and their shape can be identified with brittleness, toughness, etc.

As far as fluid characterisation is concerned, the most usual trace of shear stress against strain rate for polymer solutions is of decreasing slope ("pseudoplasticity"). Low molecular weight and swollen particulate systems sometimes give Newtonian behaviour ($\eta$ independent of $\dot{\gamma}$) or dilatancy ("shear thickening"), respectively (Fig. 2). For all such solutions it is useful to measure $\eta$ over as wide a range of $\dot{\gamma}$ as is possible. Measurement over more than 5 decades of strain rate is possible with certain sophisticated viscometers. Those that can measure both steady and oscillatory shear are sometimes referred to as 'rheogoniometers' or 'mechanical spectrometers'.

## Viscoelastic Materials

As implied above, all materials can behave as either liquid or solids, depending upon the timescale of the rheological experiment. At very long times, "solid" materials flow as liquids under a sustained stress. For example window glass is not a solid at all, but a very viscous fluid ($\eta \approx 10^{12}$ Pa s). By contrast, at very short times (or equivalently at very high frequencies - say < $10^{-8}$ s) water behaves as a solid. Therefore, practically all materials are viscoelastic. The timescale of the deformation determines the proportion of viscous (fluid) to elastic (solid) properties in a given material.

For an elastic solid, $\tau = \gamma G$; for a viscous fluid, $\tau = \dot{\gamma}\eta$; and for a simple viscoelastic material, $\tau$ depends upon both $\gamma$ and $\dot{\gamma}$. Some solutions are extremely complex so that the actual stress also depends sensitively upon the strain (and strain rate) history.

To clarify this, pseudoplasticity is not the same as "thixotropy". In thixotropy, stress is monitored as a function of time at a constant strain rate and at long times the stress tends to decrease. For very structured fluids sometimes referred to as "weak gels", the effects may be quite noticeable. The converse phenomenon "rheopexy", in which stress increases with time, is almost invariably an indication of aggregation or gelation.

## Oscillatory Measurements

We have assumed above that the stress, or strain/strain rate applied has been nonoscillatory. For example, the cone was assumed to be always rotating in the same direction. This is still the most commonly used method for characterizing both solids and liquids. However, over the last 15 years the oscillatory strain experiment has become much more widely employed.

This reflects the improvement in equipment design and computer software, which have turned such experiments from being the realm of the specialist rheologist into becoming a routine

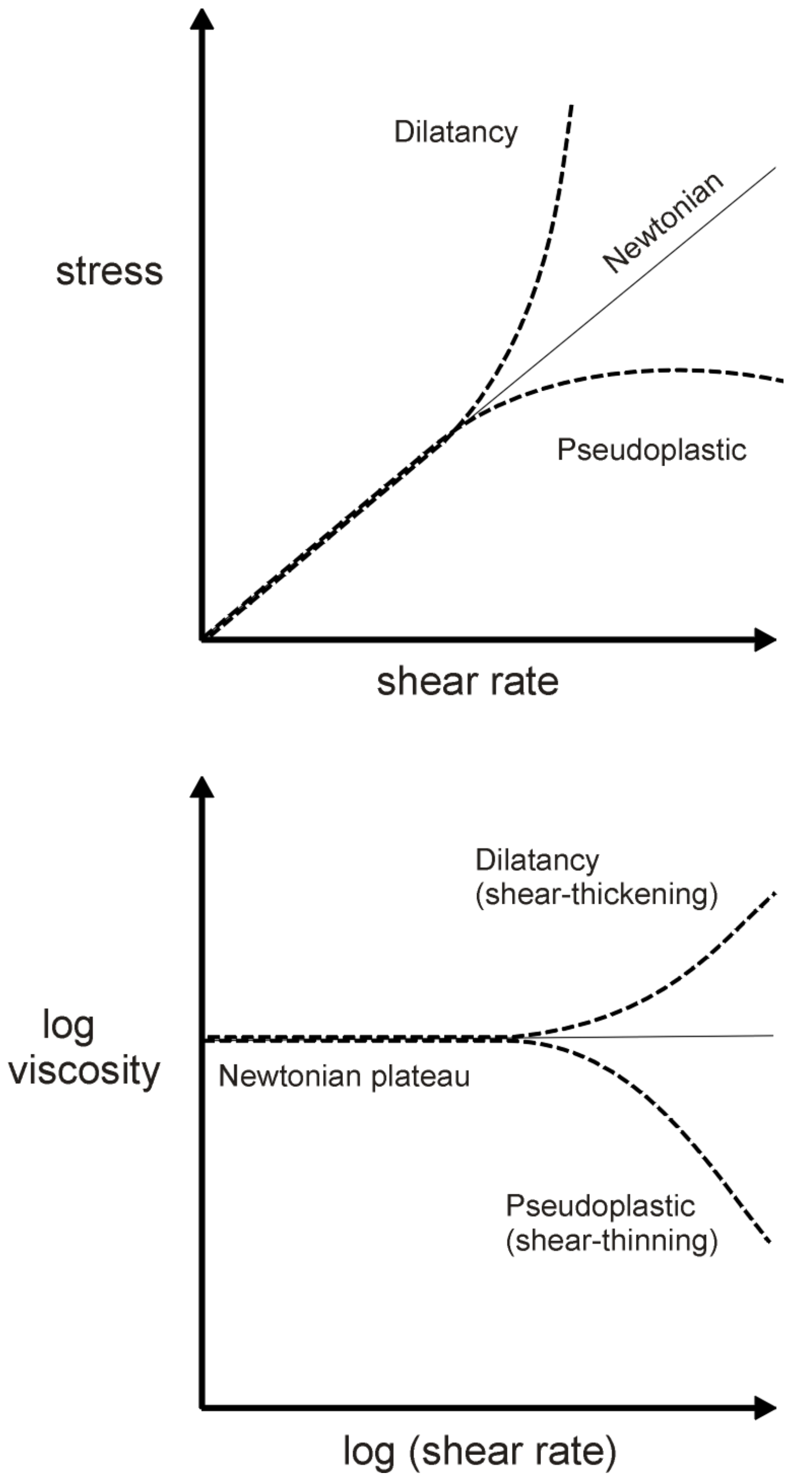

**FIGURE 2**. Flow curves for different classes of fluids.

procedure. Fig. 3 illustrates the principle — a sinusoidal oscillation of maximum strain $\gamma_M$ and oscillatory frequency ω is applied to a sample using cone-and-plate plate geometry. If the material is perfectly elastic then the resultant stress wave is exactly in phase with the strain wave. By contrast, since the strain rate is maximum when the strain is zero and vice versa, for purely viscous systems, the resultant stress wave will be exactly $90^0$ out-of-phase with the imposed

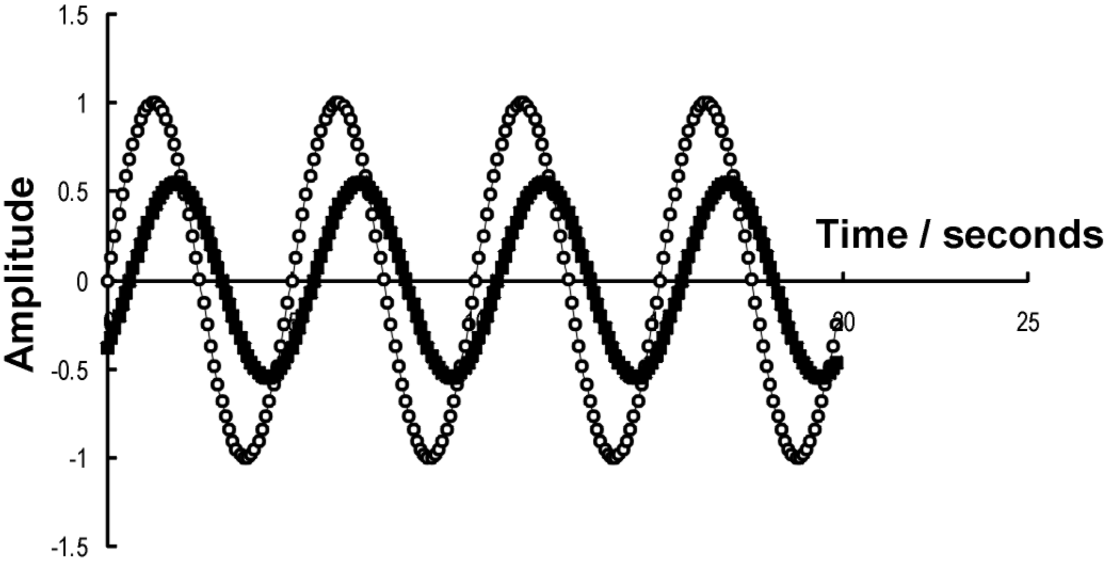

**FIGURE 3.** Oscillatory strain experiment.

deformation. In general the stress wave will have a phase difference δ ($0 < \delta < 90^0$), so that δ, or more usually tan δ is a measure of the viscous/elastic ratio for the material at frequency ω. The elastic (in-phase) and viscous (out-of-phase) components of the stress wave are separable by software correlation analysis. The in-phase, shear storage modulus G′ and the out-of-phase, shear loss modulus G″ are then defined by:

$$G^* = \tau^* / \gamma_M = (G'^2 + G''^2)^{½} \qquad (4)$$

with $G^*$ the complex shear modulus, $\tau^*$ the complex stress and $\eta^*$, the dynamic viscosity is equal to $G^* / \omega$ and tan δ is the ratio G″ / G′.

The above description is true only for very small strains. As far as viscoelastic measurements are concerned the coupling of strain and strain rate (frequency) can be a serious problem. This is because only in the small strain limit will the correct G′, G″, $\eta^*$ profiles be obtained. The design of an instrument with good minimum strain resolution and good stress detection sensitivity is a testing task. (Unfortunately the purchase of such an instrument can be quite costly.) Fig. 4 shows a typical configuration.

A number of oscillatory controlled stress rheometers have appeared on the market since they have the advantage of being perhaps half the cost of controlled strain rheometers. If the stress, τ, is constant, then the strain, γ, becomes the independent variable. There is no independent control to ensure this strain remains small. For fluids, for example, the strain will tend to increase inversely proportional to the applied frequency. This effect is less serious for more solid materials, but should still be carefully examined. In practice most stress instruments use a feedback loop to maintain the strain constant and so claim to measure G′ and G″ directly. In most cases they perform this very satisfactorily, but for precise experiments the intrinsically more expensive controlled strain type instruments do have an advantage.

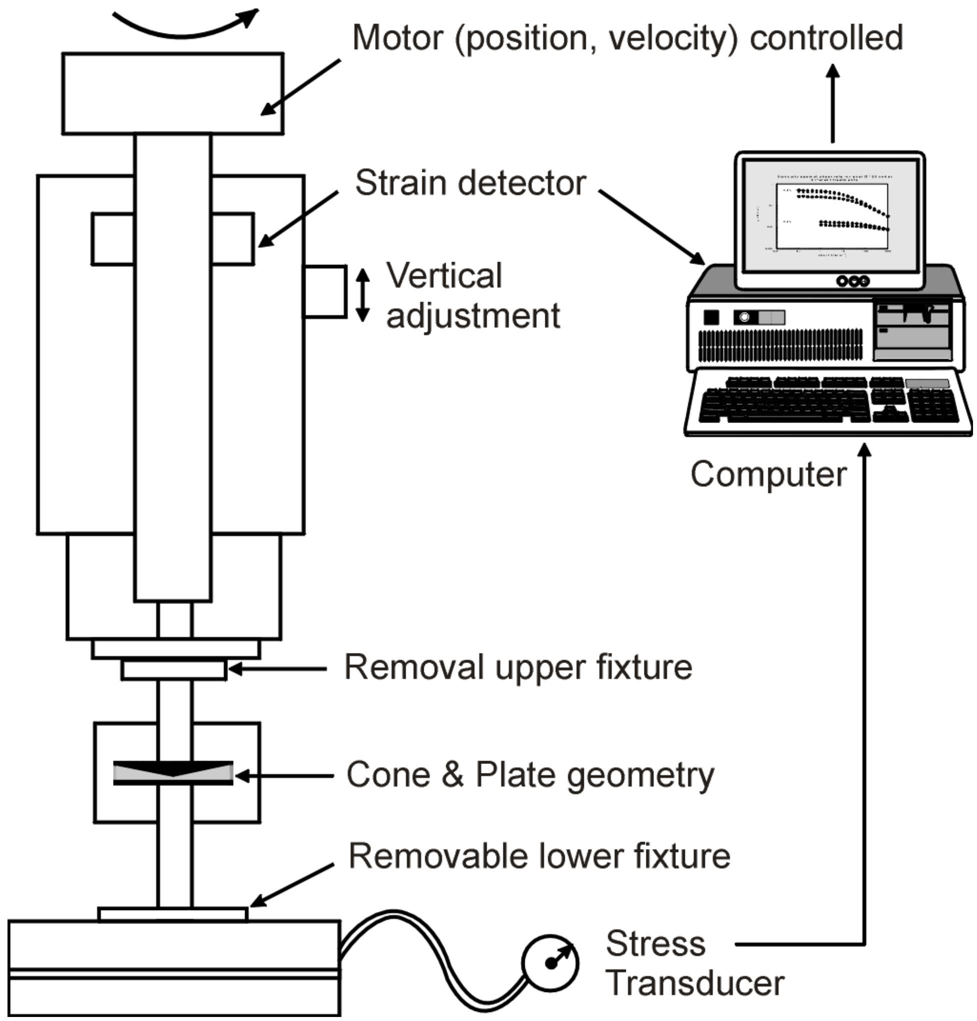

**FIGURE 4.** Schematic representation of a modern controlled strain rheometer.

## MACROMOLECULAR ASPECTS OF BIOPOLYMER RHEOLOGY

Few of the functional properties of biopolymers are directly governed by their primary sequence structure. Much more significant are the space-filling conformations adopted. The secondary structure leads to the tertiary structure, i.e., the shape of the whole macromolecule. For example, some marine polysaccharides such as carrageenan and agarose involve quite extensive double helical structures. Nevertheless, many carbohydrate polymers behave as flexible coils in dilute solution, and their solution properties may be described by the random flight model. In this the coil (root mean square) size, the radius of gyration $R_g$, is proportional to the square root of their molecular weight (relative molecular mass) $M_r$. For spherical particles, an approximation to the shape of globular proteins, the volume of the sphere $\propto R_g^3$, and thus $R_g \propto M_r^{1/3}$, while for perfectly rigid (but infinitely thin) rods $R_g \propto M_r$. Typical sizes, in terms of radius of gyration, are ~25 nm for flexible biopolymers, and >100 nm for very stiff (worm-like or rod) biopolymers.

## CONCENTRATION REGIMES

We now introduce the effect of concentration on the material properties of biopolymer solutions, in terms of a 'penetrable sphere' model representing the time average shape of the biopolymer. The volume of this equivalent sphere is $4/3\pi R_g^3$. For a solution of C kg of macromolecules per $m^3$ (= C grams per litre), there will be $CN_A/M_r$ macromolecules, where $N_A$ is Avogadro's number. The total volume occupied by the macromolecules is then $4/3\pi R_g^3 CN_A/M_r$.

It has become conventional to denote the total occupancy concentration by $C^*$ (where $C^*$ is proportional to $M_r/R_g^3$). Concentrations below $C^*$ are denoted 'dilute', and those well above $C^*$ are termed 'semi-dilute'. Here 'dilute' has a specific meaning, in terms of the space occupancy, so that whether a solution is regarded as dilute does not depend on the concentration say in weight percent. In other words a 10% solution of small particles could be dilute, and a 1% solution of larger particles could be semi-dilute.

For dilute solutions we expect to see the behaviour of isolated macromolecules. For semi-dilute solutions we have a more homogeneous view of a collection of polymer residues (segments), without knowing which segment belongs to which individual chain. It also follows that if a viscosity increment is required at low concentration, polymers that have extended coil or rod-like chain profiles will be much more effective than hard sphere or globular macromolecules.

## INTRINSIC VISCOSITY

Above we introduced the definition of a dilute solution. In this regime, the "viscosity increment" over the solvent depends upon the size/shape of small numbers of macromolecules. For solutions only just more viscous than the solvent we can estimate the macromolecular properties of an individual polymer. For example, the first "viscosity increment" rule was that of Einstein, who wrote

$$\eta = \eta_s (1 + 2.5\,\phi + ...) \tag{5}$$

where $\varphi$ is the "volume fraction" of spherical particles, $\eta$ the solution viscosity, and $\eta_s$ the solvent viscosity. Rearranging this equation, and assuming the higher terms are neglected (as $\varphi \rightarrow 0$), we have

$$(\eta - \eta_s) / \eta_s = 2.5\,\phi + .... = [\eta]_{spheres} \tag{6}$$

If the disperse phase and fluid densities are close to unity, the volume fraction is equal to the concentration in units of g $ml^{-1}$ ($10^3$ kg $m^{-3}$), and $[\eta]$ is known as the intrinsic viscosity. In practice $[\eta]$ is mostly defined in units of reciprocal percentage concentrations (g $dl^{-1}$), so that from Eq. 6 the intrinsic viscosity of small spherical particles is said to be $2.5 \times 10^{-2}$ dl $g^{-1}$.

$[\eta]$ has been calculated for solid prolate (rod shaped) and oblate (disc shaped) ellipsoids as a function of axial ratio (L/d). For (L/d) of 10, $[\eta] = 8.1 \times 10^{-2}$dl $g^{-1}$ and $13.9 \times 10^{-2}$dl $g^{-1}$ for oblate and prolate ellipsoids, respectively.

In the 1920s Staudinger proposed that for flexible synthetic polymers, the intrinsic viscosity was directly proportional to $M_r$; although this showed great insight, the hypothesis was later found not to be completely correct. Nowadays the relevant expression (the Mark-Houwink-Sakurada equation) is written as

$$[\eta] = K' M_r^{\alpha} \tag{7}$$

Here $\alpha$ = 0 for a sphere, 1.8 for a rod, and ideally 0.5–0.8 for flexible polymers in "marginal" and

"good" solvents.

The most widely used viscometer for very dilute solutions is of the glass Ostwald-Ubbelohde capillary design. This has two etched (upper and lower) marks, and the time for the solvent, $t_s$ and the solution, t to flow between these, is noted. The relative viscosity for the solution is then just the ratio $t/t_s$. Automatic timing and dilution viscometers can be obtained, and the whole glassware can be immersed in a finely controlled temperature bath (<0.05°C is really required). Other multibulb glass designs allow measurements to be made at several different shear rates and extrapolated to zero.

For many users a more conventional concentric cylinder viscometer is preferred. In this, the solution is poured into a narrow gap between two concentric cylinders (Couette geometry). One of these turns at a constant rate (the rotor), the second (the stator) is attached to a torque measuring transducer. The range of shear rates is typically 0–20 $s^{-1}$, and the sample volume can be as low as 0.5 ml. Clearly this is advantageous for examining scarce samples of biological origin.

The ratio of efflux times t and $t_s$, for solvent and solutions described above, or the slopes m, $m_s$ of the input/output voltages above, gives the relative viscosity $\eta_r$ for the solution, i.e., $\eta_r = \eta/\eta_s = t/t_s = m/m_s$. If $\eta_r$ lies in the range say 1.2–2.2, the intrinsic viscosity may be obtained as follows. The specific viscosity $\eta_{sp}$ is defined to be $(\eta_r - 1)$, so that:

$$[\eta] = \lim_{C\to 0}\left\{\eta_{sp} / C\right\} \approx \lim_{C\to 0}\left\{\ln(\eta_r)\right\} \qquad (8)$$

where the plots of $\eta_{sp}/C$ and $\ln(\eta_r)/C$ vs. C are due to Huggins and Kraemer, respectively.

Most polypeptides, and many polysaccharides are polyelectrolytes. This means that the dimensions of an individual macromolecule change with the overall ionic strength. Diluting a polyelectrolyte with water will modify the ratio $R_g^3/M_r$, as the chains will tend to expand to minimise backbone charge-charge interaction, leading to difficulties in the [η] extrapolation. In practice it is essential to dialyse the polyelectrolyte against a solution of salt of known I, and then to make the necessary serial dilutions using the dialysate.

## BIOPOLYMER SOLUTIONS AS FLUIDS

As the concentration of biopolymer is increased, differences in behaviour are seen, depending upon the nature of the biopolymer. For essentially random coil systems, entanglement networks are formed by the simple topological interaction of polymer chains rather than by cross-linking. They occur when C becomes >~ 5C*, defined above. For stiffened, or partially ordered systems, so-called structured fluids (sometimes called weak gels) result. We note here that a precise definition of the class of materials referred to as "weak gels" was given by one of the present authors[1] almost 10 years ago. Although we will continue to use this term, there is a real difference between those materials that can flow at large deformation (structured fluids) and those that cannot (solids - or in this context "true" gels). The precise behaviour depends upon the response to strain, concentration, etc., and it is convenient to distinguish between these "weak" and strong gels.

Bearing this *caveat* in mind, rheological discrimination between these classes of material can be made by the technique of dynamic mechanical analysis ('mechanical spectroscopy'). Typical 'mechanical spectra' are illustrated in Fig. 5. For entanglement networks, at low frequency $G' \approx \omega^2$ and $G'' \approx \omega^1$, while as the frequency is decreased there is a 'cross-over' in G′ and G″. At the very

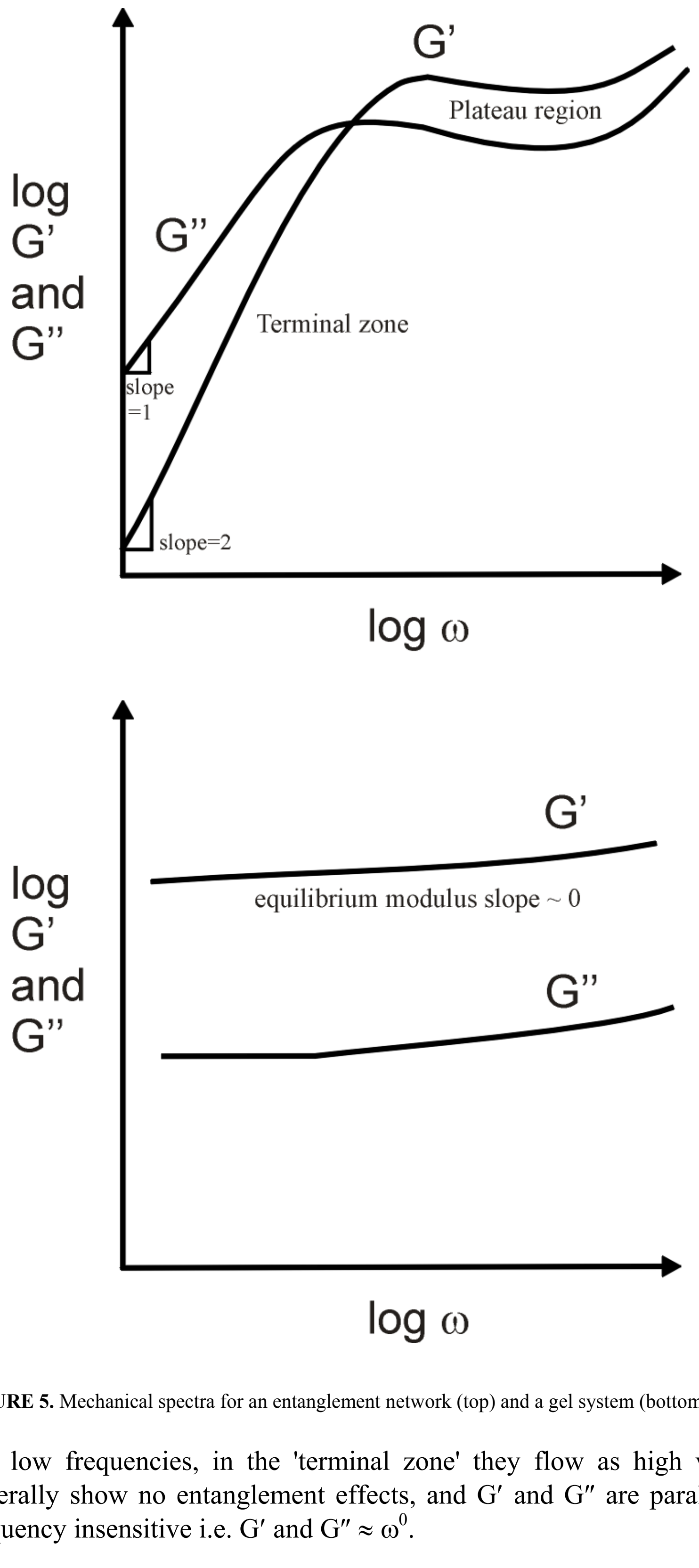

**FIGURE 5.** Mechanical spectra for an entanglement network (top) and a gel system (bottom).

low frequencies, in the 'terminal zone' they flow as high viscosity liquids. Gel systems generally show no entanglement effects, and G′ and G″ are parallel, with G′ > G″ and largely frequency insensitive i.e. G′ and G″ ≈ $\omega^0$.

## Entanglement Solutions

For entanglement solutions, well above $C^*$, the viscosity decreases increasingly strongly with shear rate, as the concentration is increased. Such behaviour is illustrated in Fig. 6 for a typical polysaccharide. The simplest explanation for the trend is that at the lowest shear rates intermolecular entanglements disrupted by the imposed deformation are replaced by new interactions between different partners, with no overall change in the extent of entanglement, and therefore in solution properties. This situation corresponds to the Newtonian region of the flow curve. The onset of more pronounced shear thinning occurs when the rate of externally imposed motion becomes progressively greater than the rate of formation of new entanglements.

For this reason it is widely accepted that the Cross equation, Eq. 9, provides a good empirical description. This has three independent parameters, $\eta_\infty$, the high shear rate viscosity (which is not generally related to $\eta_s$) m, a power-law exponent and $\lambda$, a relaxation time.

$$\eta = \eta_\infty + (\eta_0 - \eta_\infty)/[1 + (\lambda\dot{\gamma})^m] \qquad (9)$$

Fig. 6 also shows the corresponding Cross fit; and how useful the equation can be in estimating $\eta_0$, particularly when this lies below the instrument shear rate range.

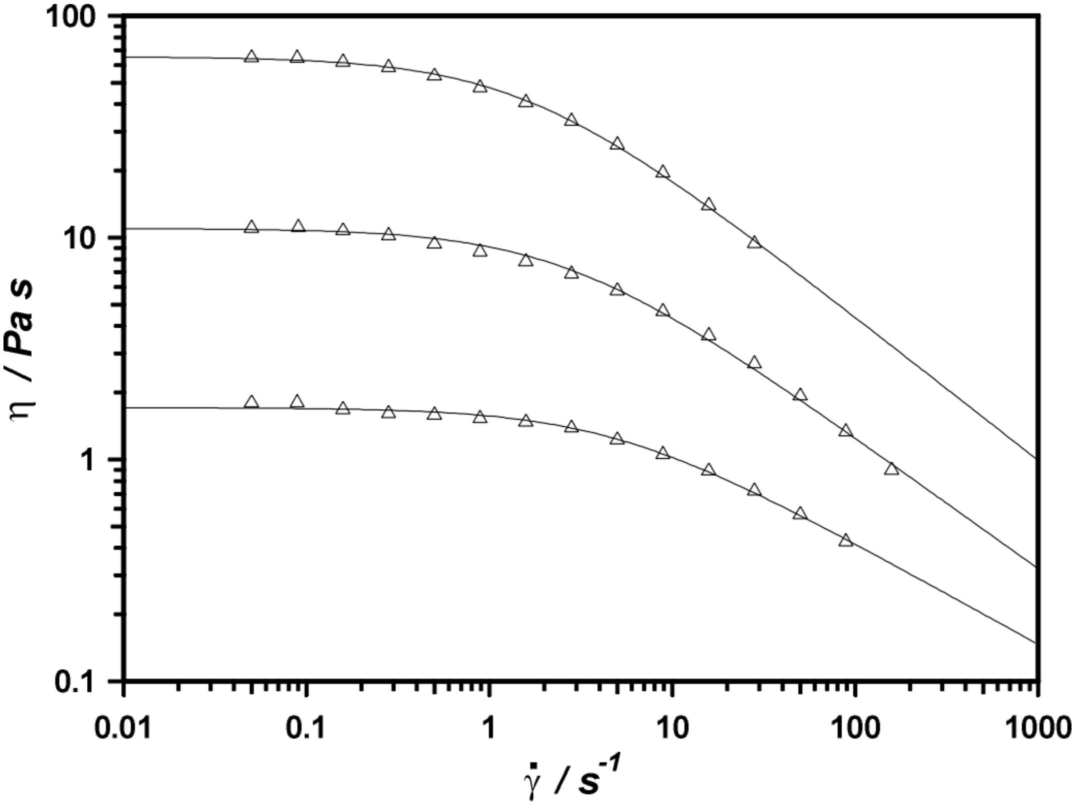

**FIGURE 6.** Flow curves for a typical polysaccharide entanglement network system.

## Structured Fluids ("Weak Gels")

Several important biopolymer systems appear to give a gel type "mechanical spectrum", with $G' > G''$ and both largely frequency insensitive, but the strain dependence of $G'$, $G''$, and $G^*$ is rather pronounced (i.e., the linear viscoelastic region usually extends only to strains of <0.1). On these, steady shear measurements can be made, although the response is very different to that seen above. Indeed, there is almost no tendency to approach a Newtonian plateau (except under very low stress conditions in a controlled stress instrument) and instead the trace of log $\eta$ vs. log $\dot{\gamma}$ is almost a pure power law, with a slope of ~ 0.8–0.9. To rheologists this is sometimes referred to as "yield stress" behaviour. Such behaviour is seen with "normal" entanglement solutions only when they contain high concentrations of dispersed particulate material. Typical examples are xanthan, schizophyllan, and scleroglucan solutions. In all cases the macromolecule is extremely stiff, tending towards rod-like. One explanation for this type of behaviour is that an element of liquid crystallinity is being developed (isotropic to nematic or cholesteric phase transition), but this is certainly not an explanation appropriate for every condition.

## The Cox-Merz Rule

For many polymer solutions, the frequency dependence of $\eta^*$ and the shear rate dependence of $\eta$ are observed to be closely superposable, when the same numerical values of $\omega$ and $\dot{\gamma}$ are compared. This empirical correlation, often called the Cox-Merz rule, has already been observed for a number of biopolymer solutions, but there are also a few exceptions. Even in these cases, useful information may be extracted from the comparison of ($\eta$, $\dot{\gamma}$) and ($\eta^*$, $\omega$) profiles. Indeed if the viscosity/shear rate profile is of the entanglement network form, with a Newtonian plateau, superposition is almost always seen. Conversely, if the weak gel "power law" behaviour described above is seen, then $\eta^*$ is almost always > $\eta$, and the difference becomes increasingly large with decreasing frequencies and shear rates. Application of the Cox-Merz rule has proved a valuable guide to characterizing the nature of structured liquids.

## Nonlinear Viscoelastic Behaviour

Earlier we discussed nonlinear effects involving both time and strain dependence, and hinted at how these are, in general, coupled for viscoelastic materials. What this means is that such measurements as those described above must be carried out at strains sufficiently small that the complex modulus $G^*(\omega)$ is demonstrably independent of $\gamma$. Unfortunately, this limiting strain depends on the nature of the system. Since the strain dependence of $G^*$ is not quantitatively predictable, it must be established by careful experiment. Fig. 7 illustrates the behaviour of typical systems. Both the polymer solution and the gel have moduli that are reasonably independent of strain up to a certain limit. On the other hand, certain materials that give a gel-like frequency spectrum at low strains, have a much more pronounced strain dependence. However, if subjected to a steady shear flow, they will apparently flow rather than fracture. Such materials, here denoted "weak gels" are exemplified by xanthan gum solutions with the product of $C[\eta] > 5$.

More significantly, as we mentioned above, such systems do not obey the Cox-Merz rule, in fact $\eta^*(\omega)$, measured in the small strain limit, lies above $\eta$ ($\gamma$), except at high frequencies, when the curves may converge. Such behaviour is usually associated with a tendency to form aggregated structures or dispersions, which are then broken down under the applied strain. A valuable method for characterizing this involves start shear and history investigations.

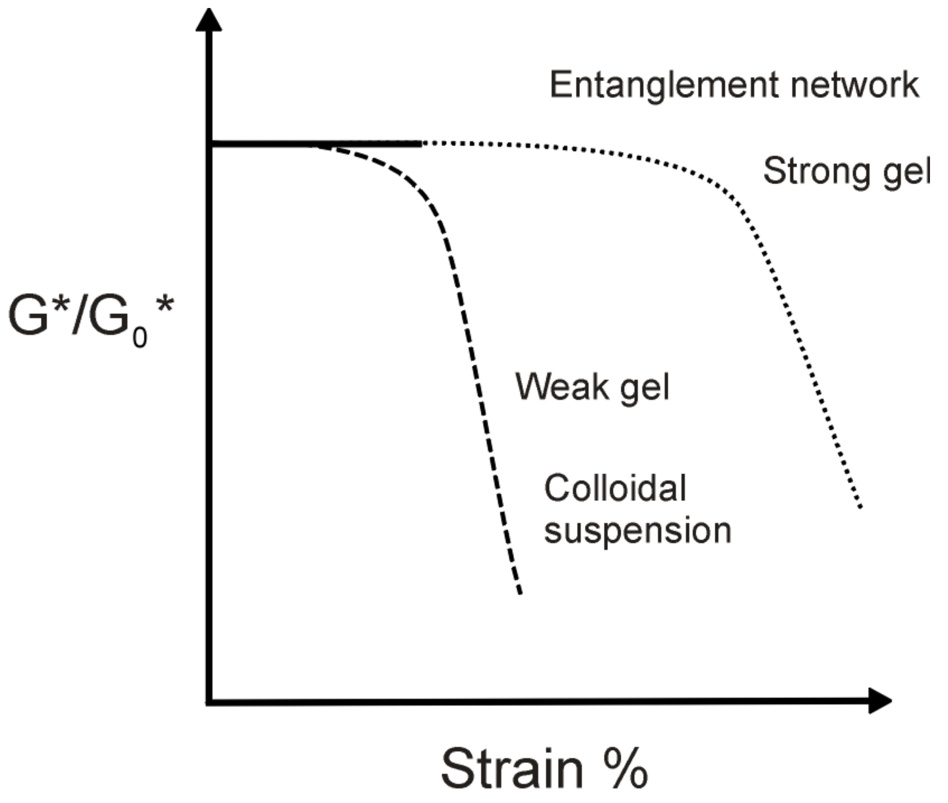

**FIGURE 7.** Strain dependence for different viscoelastic materials.

## Start Shear and History Experiments

These are the equivalent of stress-strain (not strain rate) measurements, but applied to fluids rather than deformable solids. The sample, at rest, usually in a cone-plate instrument, is "instantly" subjected to a constant deformation (shear) rate - since the "rise time" has to be less than 50 ms; a very good instrument is required. The stress developed is then monitored with time. If $\dot{\gamma}$ is constant, then $\dot{\gamma}$ t = $\gamma$ (t), and $\tau$ (t) can be plotted against $\gamma$ (t). Typically, at high shear rates the stress will rise to a maximum, $\tau_{max}$, "overshoot", before falling to the time independent stress corresponding to the steady shear viscosity (= $\tau / \dot{\gamma}$); at lower shear rates $\tau$ (t) will rise monotonically to $\tau$.

To investigate the time scale of recovery of "weak gel" (thixotropic) materials it is useful to adopt a procedure in which the sample is first extensively sheared, at constant $\gamma$ to remove all past deformation history, then allowed to rest for a given time t′, before suddenly restarting at the original constant strain rate. In this way the overshoot ratio, $\tau_{max}/\tau$ can be charted for different rest times t′. If the history effect is small, a maximum ratio will be reached after quite short t′ times, conversely for highly structured liquids it may take many hours to reach equilibrium.

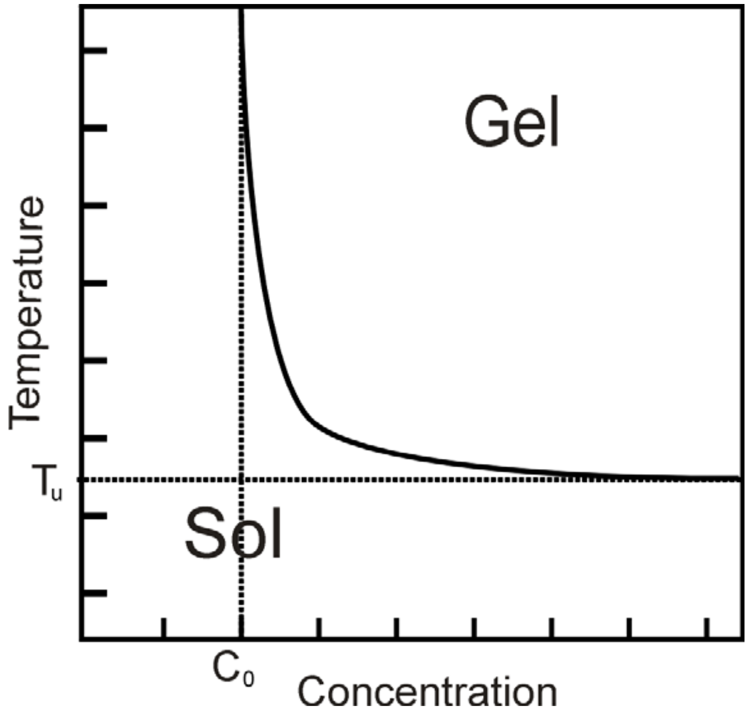

**FIGURE 8**. Temperature vs. concentration in the lower critical solution temperature (LCST) type phase diagram.

## BIOPOLYMER GELS

Making rheological measurements on biopolymer gels is not intrinsically difficult these days with efficient computer-driven instruments. For most systems that set on cooling, i.e., gelatin, agar(ose), carrageenan, pectin, gellan, etc., this suggests loading the solutions hot and allowing to cool.

By the same token, some aspects of heat-set gelation of globular protein solutions are familiar to anyone who has boiled an egg. Nevertheless, a detailed understanding of factors controlling the rate of gelation and the final “gel strength” achieved and how these depend on the implicit variables, such as the protein sequence, concentration, pH, and ionic strength, are less well established. Historically, the picture is that on denaturation a protein completely unfolds, and then intermolecular interactions lead to the formation of a fine-meshed “macromolecular” gel. This principle is still quite appropriate for gelatin and for some chemically denatured globular proteins, but for heat-set globular proteins the picture is rather different.

For example, it is now appreciated that in simple heat-induced denaturation, the protein size and shape is only mildly perturbed. Instead some of the hydrophobic groups, which at ambient temperatures remain buried in the protein core, become exposed above the unfolding, or denaturation temperature, $T_m$. This “hydrophobic effect” leads to aggregation to form either fine stranded networks, or amorphous particulate structures of a “physical gel”. At secondary structure level this usually means that there is a small decrease in the $\alpha$-helix component, and an increase in the proportion of $\beta$-sheet.

For “cold set” gels, by contrast, the high temperature form is “disordered” and ordering occurs, say by passing through a coil-helix transition, on cooling. This reversion to the native form does indeed produce a fine-stranded, often fibrillar, structured gel. Much interest in both cold and heat set gel rheology is in mapping out the sol-gel state diagram. A typical example is illustrated in Fig. 8.

## Small Deformation

Nevertheless there are a few experimental pitfalls. Firstly it is usual to perform the gelation experiment *in situ*, by filling the gap of the rheometer with solution and then gelling in the instrument.

The two main artifacts are “crusting” or “drying” of the sample at the exposed edge, resulting in a film of polymer. This can be prevented by covering with mineral oil. The second problem is “slip”. As the gel sets up it may synerese, and the instrument reading will become distorted by the lack of bonding between the gel surface and the metal surfaces of the instrument geometry. In some cases this is such a severe problem that no sensible data can be collected. Recent work by Richardson and Goycoolea[2] has pioneered the use of a special geometry that does seem to eliminate this troublesome problem.

Assuming we can obtain good data, however, for systems allowed to gel under a well-defined thermal regime, and allowed to achieve a limiting degree of cross-linking, there is a characteristic “cure” curve (a term taken from the curing of thermo-set resins) of log (G′, G″) against time. This has an initial lag time, then both G″ and G′ increase, but with G′ increasing faster than G″ giving a cross-over time (very often taken as the gelation time), and finally G′ appears to reach a plateau value (although for some gels there is actually no final value of the modulus since log [G′] appears to increase indefinitely when plotted against the log of time). G″ sometimes passes through a parabolic maximum and then decreases to zero, and sometimes plateaus off. We have observed and noted a qualitative correlation, with biopolymer gels formed from more flexible chains giving such a G″ parabola, whereas those from stiffer chains (e.g., globular proteins, agarose) give a slight maximum followed by a plateau.

From cure curves measured for different initial polymer concentrations, characteristic (long time) modulus-concentration and gelation time-concentration dependencies are found. For example, for the modulus G, near the critical gel concentration $C_0$ (the concentration below which no gel can be formed), a large and variable power law dependence of G on C is observed, whilst at much higher concentrations, a constant, and approximately $C^2$, limiting law emerges. Such behaviour has been noted historically. In more recent literature, some data appear to fall closer to the asymptotic scaling prediction $G \propto C^{2.25}$ (although discrimination between these values is quite difficult).

## Large Deformation

Large deformation testing involves examining a sample either in tension/compression or shear, up to and sometimes past the yield or failure point. While measurements for example for rubbers are usually quite simple to perform, for some low modulus gels and networks there are experimental difficulties in obtaining meaningful data.

Many workers have taken the easy option, by preparing and then compressing cylindrical samples. However in such an experiment the response is often dominated by friction between the sample surface and the compressing plates and the sample begins to “barrel” or “bulge”. If the Poisson's ratio of the gel is close to 0.5 (totally incompressible) uniaxial compression in one plane is equal to biaxial extension in the other two. Such biaxial extension can only occur if the surfaces of the specimen are very well lubricated. Tensile measurements are just as difficult to perform because merely gripping weaker gel samples with pneumatic or screw clamps will induce preferential failure at the clamping point. Large deformation measurements in shear are also difficult to perform because debonding of the sample from the test geometry is very difficult to avoid. This can be reduced by roughening the fixture surfaces, but then calculation of stresses and strains is made difficult.

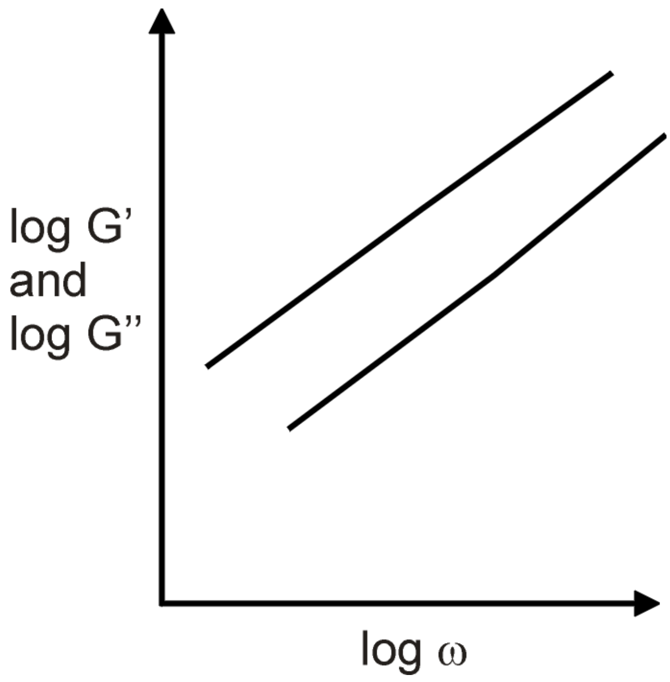

**FIGURE 9.** Mechanical spectra showing the common power-law dependence of G′ and G″ at the gel point.

Christianson and coworkers[3] have compared the large deformation behaviour of gels under both lubricated and unlubricated conditions in uniaxial compression. The lubricated surfaces typically require paraffin oil and Teflon covered platens, while the unlubricated experiment uses cyanoacrylate adhesive. Alternative experimental methods, which have proven valuable, include extending either rings or, even better, "hippodrome" or "race track" shape gel samples over dowel pins. Then tensile measurements can be made without the need to use clamps or measuring cylindrical gel samples in tension, by supergluing the ends on to roughened supports, themselves attached to clamped metal plates.

## Critical Gels

For a long time, it was thought to be difficult (if not impossible) to determine the exact gelation point by rheological methods, the incipient network is extremely tenuous, and it would require an infinite time for the gelling system to relax to equilibrium. However, many authors have tried to use dynamic measurements to assess what we may refer to as the rheological gel point. For example, it may be judged to take place when the sample gelling signal becomes just greater than the background noise, when say (the shear storage modulus) becomes greater than a preassigned threshold value. Alternatively, it can be when G′ becomes greater than G″, the shear loss modulus (i.e., when tan δ becomes just less than 1) sometimes called the cross-over point. The method of Winter and Chambon[4] helps provide a sounder approach. They asserted that at the gel point both $G'(\omega)$ and $G''(\omega) \sim \omega^n$ (Fig. 9). Theoretical estimates for the exponent lie mostly in the range 0.5–0.75; corresponding experimental results for a number of systems fall mainly in the range 0.4–0.8

## CONCLUSION

This summary represents a brief overview of the area of biopolymer rheology. It has deliberately adopted a “nonsystem specific” viewpoint, and consequently been unable to deal with particular cases in any real detail. The references given below are of general interest, since there are few monographs in this area, nevertheless they should provide some background material for what is an ever-increasing area of importance.

## ADDITIONAL REFERENCES

Clark, A.H. and Ross-Murphy, S.B. (1987) Structural and mechanical properties of biopolymer gels. *Adv. Polym. Sci.* **83**, 57–192.

Ferry, J.D. (1980) *Viscoelastic Properties of Polymers*. 3rd ed. John Wiley & Sons, New York.

Lapasin, R. and Pricl, S. (1995) *Rheology of Industrial Polysaccharides: Theory and Applications*. Blackie Academic and Professional, London.

Launay, B., Doublier, J.L., and Cuvelier, G. (1986) In *Functional Properties of Food Macromolecules.* Mitchell, J.R. and Ledward, D.A., Eds. Elsevier Applied Science, Amsterdam. pp. 1–77.

Morris, E.R. (1984) In *Gums and Stabilizers for the Food Industry 2*. Phillips, G.O., Wedlock, D.J., and Williams, P.A., Eds. Pergamon, Oxford, pp. 57–78.

Morris, E.R. and Ross-Murphy, S.B. (1980) *Tech. Carbohydr. Metab.* **B310**, 1–46.

Ross-Murphy, S.B. (1984) In *Biophysical Methods in Food Research.* Critical Reports on Applied Chemistry. Vol.5. Chan, H.W.-S., Ed. SCI Blackwell, Oxford. pp. 138–199.

Ross-Murphy, S.B. (1994) In *Physical Techniques for the Study of Food Biopolymers*. Ross-Murphy, S.B., Ed. Blackie Academic and Professional, London. pp. 343–392.

Ross-Murphy, S.B. (1995) In *New Physico-Chemical Techniques for the Characterization of Complex Food Systems*. Dickinson, E., Ed. Blackie Academic and Professional, London. pp. 147–156.

Picout, D.R. and Ross-Murphy, S.B. (2001) Thermoreversible and irreversible physical gels from biopolymers. In *Polymer Gels and Networks.* Osada, Y. and Khokhlov, A.R., Eds. Marcel Dekker, New York. pp. 27–46.
